# Development of public stigma toward people with mental health problems in Czechia 2013–2019

**DOI:** 10.1192/j.eurpsy.2021.2226

**Published:** 2021-08-16

**Authors:** Petr Winkler, Tomáš Formánek, Karolína Mladá, Sara Evans Lacko

**Affiliations:** 1 Department of Public Mental Health, National Institute of Mental Health, Klecany, Czechia; 2 Health Service and Population Research Department, Institute of Psychiatry, Psychology and Neuroscience, King’s College London, London, United Kingdom; 3 EpiCentre, Department of Psychiatry, University of Cambridge, Cambridge, United Kingdom; 4 Department of Psychiatry, Faculty of Medicine in Pilsen, Charles University, Prague, Czechia; 5 Care Policy and Evaluation Centre, London School of Economics, London, United Kingdom

**Keywords:** Anti-stigma program, mental health care reform, mental health literacy, public attitudes, stigma

## Abstract

**Background:**

We aimed to assess the changes in public stigma towards people with mental health problems in Czechia; and to investigate the association between these and the exposure to the ongoing mental health care reform and one of its implementation projects focused on reducing stigma.

**Methods:**

We analyzed data from three cross-sectional surveys representative of the Czech adult population. We used linear regression models to compare population attitudes and desire for future contact with people with mental health problems between the 2013/2014 baseline and the 2019 follow-up. In our 2019 sample, we employed linear regression models to assess the relationship between exposure to mental health care reform and nation-wide anti-stigma campaign, and population stigmatizing attitudes and intended behavior. We utilized a propensity score matching procedure to mitigate potential bias.

**Results:**

The 2013, 2014, and 2019 datasets consisted of 1797, 1810, and 1077 participants, respectively. Population attitudes improved significantly between 2014 and 2019 (*B* = 0.99, 95% CI = 0.06; 1.93), but we did not detect a change in population desire for future contact with people with mental health problems. Exposure to the nationwide anti-stigma campaign or mental health care reform was associated with more favorable attitudes (*B* = 4.25, 95% CI = 2.07; 6.42 and *B* = 7.66, 95% CI = 3.91; 11.42), but not with higher desire for future contact with people with mental health problems.

**Conclusions:**

Mental health care reform and its nation-wide anti-stigma project seems to have a positive impact on population attitudes, but not on desire for future contact with people with mental health problems.

## Introduction

Stakeholders in central and eastern Europe (CEE) consider high levels of public stigma a major obstacle for mental health care development in their countries [1]. However, empirical studies assessing levels of public stigma in the region of CEE are scarce [1,2], and those which compared CEE with other European countries reported inconsistent outcomes. A study focused on attitudes toward people with mental illnesses in Germany, Bratislava (Slovakia), and Novosibirsk (Russia) did not find significant differences in population attitudes across the three settings [3]. Another study comparing attitudes toward people with depression in Germany, Hungary, Ireland, and Portugal found worse personal stigma in Hungary than in any of the other participating countries; but perceived stigma was similar across these countries [4]. A study that assessed the reported and intended behavior toward people with mental health problems showed that both are considerably more favorable in England than in Czechia [5]. In addition, the community attitudes toward people with mental illness pointed out to an unusually high level of stigmatizing attitudes in the Czech population; however, a similar study from neighboring Slovakia demonstrated notably better population attitudes there [6,7].

Similarly, in terms of experienced or anticipated stigma, the INDIGO study, which used small and not representative samples, found no clear differences between western European and CEE countries in terms of discrimination experienced by people with psychosis [8]. However, the FEDORA study found people with a first episode of schizophrenia or depression reporting discrimination more frequently in Poland and Sweden than in Croatia, Czechia, Romania, or Turkey [9]. Another study identified higher internalized stigma in people with mental disorders in Croatia, Lithuania, and Romania when compared to Sweden, but not higher in comparison to Malta; and the same study identified higher perceived devaluation and discrimination in Sweden, Lithuania, and Croatia than in Malta or Romania [10].

The communist regime, which prevailed in CEE in the second half of the 20th century, has profoundly influenced mental health care systems in the region [1,11]. Based on ideological reasons, the communist regime supported asylum-like psychiatric hospitals and excessively long (often life-time) hospitalizations of people with severe mental illnesses, encouraged stigmatizing attitudes, centralized decision making and prevented service users and families to take part in it, supported biologically oriented psychiatry, and discouraged a public mental health approach [11,12]. In the Czech Republic, several initial attempts to transform mental health care were pursued after the collapse of the communist regime in 1989; however, similar to other countries in the region, these attempts were not very successful [12,13]. Mental health care remained institutionalized [14], economically ineffective [15,16], and problematic from a human rights perspective [17]. Attitudes toward those with mental illness have been unfavorable among the general population as well as among medical doctors or students [5,6,18], and people with mental health problems and their relatives often feel that they are targets of stigma and discrimination [19,20].

The Czech Republic is one of the very first CEE countries, which launched a government-backed mental health care reform. In 2013, the Czech Ministry of Health published the Strategy of Mental Health Care Reform [21], which aims to improve the quality of life of people with mental health problems. The strategy is implemented through 10 implementation projects, which started between 2017 and 2019, and which include the following: Three projects entitled “Community Mental Health I, II and III” focused on establishment and operation of new types of community services which combine health and social care, and which are correspondingly financed from both, health insurance and social care budgets. The project Deinstitutionalization is focused on transforming psychiatric hospitals, improving quality of care as well as adherence to the Convention on Rights of People with Disabilities, and on decreasing a number of long-term hospitalizations. The project Multidisciplinarity aims to encourage the best practice in service provision via educating and training health and social care professionals. The project New Services is a pilot of community mental health teams for children and adolescents, forensic patients, people with substance use problems, and people with dementia. The project entitled Destigmatization was launched in-line with international recommendations [22,23], and it is described more in detail below. The project Early Detection and Early Intervention Services is dedicated to adopting and piloting timely intervention services for people at risk of psychosis or those with the first episode of psychosis. Finally, the project Strengthening Evidence-based Mental Health Care Development and the project Analytical Support for Mental Health Care Reform are both focused on supporting evidence-based mental health care development. In 2020 and 2021, National Mental Health Action Plan, National Suicide Prevention Plan, and National Action Plan for Alzheimer’s and Other Dementias 2030 were approved by the Czech government, which secures the continuity of mental health care reform as well as its extension into the field of prevention and promotion. Since the start of the mental health care reform implementation projects, the mental health care reform is often cited and presented in national media outlets.

We aimed to assess whether people exposed to the mental health care reform or to a nation-wide anti-stigma project report more favorable attitudes and lower desire for social distance toward people with mental health problems than those who were not exposed to such initiatives. In addition, we aimed to assess the changes in stigmatizing attitudes and behavior in Czechia between 2013/2014 and 2019.

## Methods

### Anti-stigma project “On the Level”

The Czech anti-stigma program and its initiative “On the Level” started in September 2019. On the Level utilizes a strategic targeted approach and focuses on six target groups: (a) health care professionals, (b) social care workers, (c) public administration workers, (d) communities living close to a mental health facility, (e) people with mental illnesses, and (f) their families. The anti-stigma work with communities was implemented mainly via the activities of the festival “Na Hlavu” (Head on Mental Health) and via the support of local anti-stigma initiatives that take place within the “Mental Health Weeks.” Mental Health Weeks is an annual event organized by mental health care providers, and it usually contains public lectures, musical concerts, art exhibitions, discussions, and displaying the work of mental health care providers. This event started more than 25 years ago and it is now organized all over the country during September and October. “Na Hlavu” is a multimedia festival, which includes the following activities: (a) movie projections, theater or other performances that relate to mental health and that are followed by discussions with mental health professionals, service users, and occasionally with artists themselves; (b) a discussion entitled “My name is not a diagnosis” which is a moderated discussion with three to five people with experience of mental health problems; (c) a lecture “Czechs and their mental health” which gives an overview of mental health problems in the Czech Republic as well as an overview of the Czech system of mental health care, including its reform; (d) the mental health seesaw, which points out to the unbalances in mental healthcare, and which promotes interactions about mental health between the two people sitting at the opposite ends; (e) an exhibition about mental health myths and stereotypes; and (f) mental health promotion activities (for instance, via interactive quizzes, leaflets, and other information materials).

### Data and participants

In the present study, we utilized data from three studies: (a) the 2013 study containing the RIBS scale, (b) the 2014 study containing the CAMI scale, and (c) the 2019 follow-up study containing the RIBS and CAMI scales. The baseline datasets are described in detail elsewhere [5,6]. In brief, the 2013 RIBS data were obtained through an omnibus survey that utilized face-to-face interviewing, conducted by a professional data collection agency. Random route sampling in randomly selected voting districts was employed. The dataset consisted of 1797 (response rate [RR] = 86%) individuals. Similarly, the 2014 CAMI data were collected in an omnibus survey that employed face-to-face interviewing and quota sampling, and was realized by a professional data collection agency. The dataset contained 1810 participants (RR = 82.1%).

The 2019 follow-up data were collected by a professional data collection agency, using a face-to-face omnibus survey that utilized a quota sampling technique to enroll potentially eligible participants. The data were collected using a mixed paper-and-pencil interviewing (PAPI, 70% of the sample) and computer-assisted personal interviewing (CAPI, 30% of the sample) technique. The RR was 59% and overall 1077 individuals were enrolled in the study.

For all three samples, data are representative of Czech noninstitutionalized adult population (aged 18 years or more) in terms of age, gender and place of residence. Post-stratification weights were applied to ensure also the representativeness of all samples as with respect to the level of education. We obtained ethical approval from the Ethical Committee of the National Institute of Mental Health, Czech Republic.

### Measures

We used the shortened 27 item version of the CAMI to assess respondents’ attitudes toward people with mental illness. The CAMI and its shortened version are considered to have high internal and external validity and acceptable internal consistency, respectively [24–26]. The shortened version of CAMI contains 13 unfavorably and 14 favorably oriented items which are rated on a scale from 1 (strong agreement) to 5 (strong disagreement). We reversed positive items so that a higher score indicated less stigmatizing attitudes and a total score ranged from 27 to 135 points.

We used RIBS to assess respondents’ past contact (i.e., reported behavior subscale of the RIBS) and their desire for future contact with (i.e., the intended behavior subscale of the RIBS) people with mental illness. The RIBS is considered to have moderate to substantial test–retest reliability and substantial internal consistency [27]. The instrument contains questions on living with, working with, having a neighbor, and continuing relationship with someone with mental health problems. While the first part of the questionnaire asks these questions with respect to the past behavior (i.e., past contact), the second part asks these questions with respect to future intended behavior (i.e., desire for future contact). The items on the intended behavior subscale are rated on a scale ranging from 1 (“strongly agree”) to 5 (“strongly disagree”). In-line with the instrument’s guideline, we assigned value 3 to the “do not know” response option [28]. Then, we summed the individual items so the RIBS score ranged from 4 to 20, with lower values indicating a more positive direction. The items belonging to the reported past behavior subscale are not included in the final score and are used only to assess the prevalence of the listed behavior. In order to assess the prevalence of past behavior, we merged the “do not know” answers on the subscale with “no” responses. In addition, we assessed the change in proportion of “do not know” responses on baseline and 2019 follow-up, comparing the combined “yes” and “no” responses with “do not know” answers.

In order to assess the exposure to the mental health care reform, we asked respondents the following question: “In the previous 12 months, did you encounter any news about the so-called reform of mental health care?,” while to assess the exposure to the nation-wide anti-stigma project, we asked the respondents whether they encountered the visual of either, the Head on Mental Health festival or the Mental Health Weeks in the previous 12 months. If an individual responded positively to at least one of the visuals, we considered him as being exposed to the nation-wide anti-stigma programme. Moreover, we employed the Level of Contact (LoC) report [28], a list consisting of 12 situations, in which closeness of contact with people with mental disorders is assessed. It ranges from “I have never observed a person that I was aware had a severe mental illness” to “I have a severe mental illness,” and the rank of the closest contact is used as the LoC score.

### Statistical analyses

We computed descriptive statistics of the sample, expressed as counts and percentages (%) for categorical variables and as means with standard deviations (SD) for continuous variables. We assessed the differences in prevalence of past behavior on baseline and the 2019 follow-up using chi-square tests. We utilized linear regression models to assess the change between the baseline attitudes and baseline intended behavior and the 2019 follow-up. We fitted crude models and models adjusted for age, gender (with men coded as reference category), and dummy coded level of education (individuals having less than a high school education considered as reference category). Next, using the 2019 dataset, we employed linear regression models to examine the association between exposure to (a) a nation-wide On the Level campaign and (b) the reform of mental health care and attitudes and desire for future contact. To control for potential bias stemming from the fact that respondents reporting campaign or reform exposure may have been more likely to have more positive attitudes and/or desire for future contact, we performed a propensity score matching. Propensity scores were calculated based on age, sex, dummy coded level of education, dummy coded size of region of residence (individuals from settlements with less than 100000 individuals as reference category), dummy coded job status (individuals not working or not pursuing own business considered as reference category) and the LoC report. We utilized the nearest neighbor method to match exposed individuals with their unexposed counterparts. To correct for matching imperfections, we included all variables (with the exception of LoC) used for propensity score calculation also in the linear regression models. The results of linear regression models are expressed as unstandardized beta coefficients (*B*) with 95% confidence intervals (95% Cis). Associations with *p* < 0.05 were considered as statistically significant. All analyses were performed in R statistical programming language (version 3.6.0).

## Results

### Participants’ characteristics

The detailed descriptive statistics of each of the survey samples are provided in Table 1.

**Table 1.** Characteristics of all samples—Czech Republic 2019 (CAMI and RIBS), Czech Republic 2014 (CAMI), and Czech Republic 2013 (RIBS).

	Czechia (RIBS) 2013 Baseline	Czechia (CAMI) 2014 Baseline	Czechia 2019 Follow-up
Total, *n*	1797	1810	1077
Gender, *n* (%)			
Women	923 (51.36)	931 (51.44)	578 (53.67)
Age, mean (SD)	45.88 (17.67)	45.80 (17.35)	48.04 (16.41)
Education, *n* (%)			
High school or higher	1076 (59.88)	1044 (57.68)	591 (54.87)
Work status, *n* (%)			
Working or entrepreneur	1094 (60.88)	NA	646 (59.98)
Size of region of residence			
100000+	404 (22.48)	NA	284 (26.37)

### Reported past contact in 2013 and 2019

When compared to the 2013 baseline using chi-square test, participants in the 2019 follow-up reported less often to live (226 [12.67%] and 77 [7.66%]), work (243 [12.87%] and 99 [8.73%]), or be a neighbor of someone (280 [14.89% and 138 [12.09%]) who has a mental health problem. We detected no statistically significant differences in relation to have a close friend with a mental health problem (297 [15.28%] and 170 [16.02%]). The proportion of “do not know” responses decreased significantly on each item of the subscale: (a) living with someone 125 (7.54%) and 14 (1.13%), (b) working with someone 185 (10.84%) and 21 (1.58%), (c) having a neighbor 274 (15.85%) and 41 (3.77%), and (d) having a close friend 179 (10.6%) and 25 (1.76%).

### Public stigma in 2013/2014 and 2019

Using a linear model, adjusted for age, gender, and dummy coded level of education, we have identified that the individuals in the 2019 sample demonstrated a lower level of stigmatization when compared to the 2014 sample (*B* = 0.99, 95% CI = 0.06; 1.93). We found no statistically significant improvement in intended behavior between baseline (2013) and 2019 follow-up (*B* = −0.03, 95% CI = −0.31; 0.25). The detailed models, including crude estimates are provided in Table 2.

**Table 2.** Linear regression models on differences in stigmatizing attitudes and intended behavior between 2013/2014 baseline and 2019.

	Intended behavior (RIBS)		Attitudes (CAMI)	
	Crude model	Fully adjusted model	Crude model	Fully adjusted model
Age	NA	0 (−0.01; 0.01)	NA	0.03 (0; 0.06)*
Gender				
Men	Reference	Reference	Reference	Reference
Women	NA	−0.38 (−0.65; −0.11)*	NA	2.08 (1.17; 2.99)**
Education				
Less than high school	Reference	Reference	Reference	Reference
High school or higher	NA	−0.48 (−0.75; −0.20)**	NA	1.52 (0.6; 2.44)**
Year				
2013	Reference	Reference	NA	NA
2014	NA	NA	Reference	Reference
2019	−0.06 (−0.34; 0.22)	−0.03 (−0.31; 0.25)	1.09 (0.15; 2.03)*	0.99 (0.06; 1.93)*

*Note:* *p < 0.05; ** *p* < 0.001. The results are expressed as unstandardized beta coefficients with 95% CIs. Post-stratification weights were applied to the analysis.

### Association between exposure to anti-stigma programme or to mental health care reform with stigmatizing attitudes and desire for future contact

The propensity score matching procedure resulted in a sample of 240 individuals exposed to the nation-wide anti-stigma campaign and 240 matched unexposed counterparts; and in a sample of 92 individuals exposed to the Czech mental health care reform and 92 matched unexposed counterparts, respectively. We found that both, exposure to the nation-wide anti-stigma campaign and exposure to the mental health care reform was associated with higher scores on the CAMI scale (*B* = 4.25, 95% CI = 2.07 and 6.42; B = 7.66, 95% CI = 3.91; 11.42), indicating less stigmatizing attitudes. For the desire for future contact, individuals who were exposed to nation-wide anti-stigma projects or the reform of mental health care did not statistically differ from those who were not exposed (*B* = −0.43, 95% CI = −1.15; 0.29 and *B* = −1.02, 95% CI = −2.31; 0.26, respectively). The details results of linear regression model are provided in Table 3.

**Table 3.** Linear regression models—the association between exposure to nation-wide anti-stigma campaign or mental health care reform and stigma-related attitudes and intended behavior.

	Attitudes (CAMI)		Intended behavior (RIBS)	
	Beta (95% CI)	Beta (95% CI)	Beta (95% CI)	Beta (95% CI)
Exposed to nation-wide destigmatization campaign				
No	Reference	NA	Reference	NA
Yes	4.25 (2.07; 6.42)*	NA	−0.43 (−1.15; 0.29)	NA
Exposed to the reform of mental health care				
No	NA	Reference	NA	Reference
Yes	NA	7.66 (3.91; 11.42)*	NA	−1.02 (−2.31; 0.26)

*Note:* *p < 0.001. The results are expressed as unstandardized beta coefficients with 95% CIs. The models are based on cases and controls matched via propensity scores. The number of individuals in models with variable on anti-stigma campaign is 480 (240 cases, 240 controls), while the number of individuals in models with variable on reform of mental health care is 184 (92 cases, 92 controls). All models are adjusted for age, gender, level of education, work status, and size of region of residence.

## Discussion

Following the introduction of mental health reform and the associated anti-stigma programme, we found that the Czech general adult noninstitutionalized population demonstrated marginally more favorable attitudes toward people with mental disorders. Individuals exposed to the nation-wide anti-stigma campaign or to the mental health care reform displayed less stigmatizing attitudes, however, not stronger desire for future contact with people with mental health problems. The finding of improvement in attitudes but not in desired future contact is in-line with a study investigating 10-year trends in public attitudes across England 6 years before and 4 years after the launch of the Time to Change anti-stigma campaign [29]. Our findings also correspond to those reported with respect to the effect of OBERTAMENT campaign in Catalonia, Spain, which suggested more favorable attitudes and slightly stronger desire for future contact among those reached by the campaign [18]. In Sweden, positive change was seen in both, attitudes and desire for future contact, and it seems that the national campaign entitled Hjärnkoll played a major role in these improvements [30]. Although there are other comprehensive nation- or region-wide campaigns in Europe, such as One of us in Denmark [31], Samen Sterk Sonder Stigma in the Netherlands, 3 Salut Mental in Balearic Islands or ENCONTRAR+SE in Portugal, peer-reviewed publications on the evaluations of these programs are not yet available or they pertain only to population subgroups, such as students [32]. Globally, the available evidence suggest that long lasting region- or nation-wide campaigns are effective in improving public mental health awareness, attitudes or discrimination toward people with mental health problems [33–35].

Although the change in attitudes is small, we believe it is important for two reasons. Similar to some other nation-wide campaigns, such as Opening Minds in Canada [36], the Czech anti-stigma campaign is primarily focused on specific target groups rather than on the general population. Secondly, as previously suggested, social representation of mental illness was negatively shaped by 20th century communism, which drove people with mental illnesses into asylum-like psychiatric hospitals so that they were hidden in front of the public’s eyes. Admitting mental disorder might have led to a life-long hospitalization, and we think that this still has a powerful influence on people’s perception of mental illness.

The association between exposure to mental health care reform and more favorable attitudes toward people with mental illness as well as the improvement in attitudes between the baseline and follow-up seems to be important, since it might help to explain higher levels of public stigma reported in countries that have not undergone deinstitutionalization yet, such as Hungary [4]. Public stigma together with institutionalization of mental health care might then function as vicious cycle, when the former reinforces the latter, and vice versa. However, more evidence is needed to support such a hypothesis, and we recommend to assess the baseline levels of public stigma in countries that are currently about to deinstitutionalize their mental health care, especially those in CEE and Central Asia [1,11]. We think that efforts to reform mental health care systems and anti-stigma programs should be considered together, because deinstitutionalization and improvements in quality and availability of mental health care services inevitably positively influence population perception of mental illnesses and their treatment; and vice versa, efforts to socially exclude people with mental health problems, for instance through institutionalization, inevitably negatively influence population perception of mental illnesses and their treatment.

Somewhat unexpectedly, and perhaps counter intuitively, we found that there was a decrease in past reported contact in 2019, as measured by the RIBS reported behavior subscale. While one explanation for this observation is worrying, indicating that people are more reluctant to disclose their mental health status or problems, another one could be related to a positive change, suggesting that the population is better at differentiating between normal reactions to everyday stresses and signs of mental health problem. While at the present moment we cannot conclusively establish the source of this change, the latter notion is partially supported by the substantially decreased proportion of “do not know” answers in the 2019 survey as compared to the baseline.

While this study has several strengths, such as the use of nationally representative samples, established outcome measures, and good RR across all of the samples; it also has a number of limitations. First, there could be a risk of social desirability bias, meaning that it is undistinguishable whether the change in attitudes was only a function of a perception of decreased social acceptability toward stigmatization or whether it reflects an authentic change in public attitudes toward people with mental health problems. In addition, since the analyses were based on a cross-sectional survey, we cannot determine the within-individual changes in attitudes and the causal pathway of these changes. However, with respect to the aims of this study, repeated cross-sectional surveys are a vital alternative to cohort studies, since they avoid attrition bias. In addition, despite using propensity score matching, we cannot entirely rule out a risk of confounding, that is, the fact that some people were exposed to mental health care reform or to a nation-wide anti-stigma program might be a function of their unobserved characteristics which is further associated with more favorable attitudes. Next, the RR in the 2019 survey was lower by more than 20%, when compared to the 2013 and 2014 surveys. While the achieved 60% RR is by standards of similar surveys still reasonable, we cannot rule out the possibility that part of the results is due to selection bias. Further, while we were able to establish whether an individual was in contact with a nation-wide anti-stigma campaign or with the mental health care reform, we do not have any information on the level of exposure and/or familiarity with these. Therefore, we were not able to investigate possible dose–response relationships between exposure and attitudes. Finally, we were not able to control for prior-to-exposition attitudes toward other minority groups, such as ethnic minorities, sexual minorities, or immigrants; which would further reduce possible bias.

In summary, this study points toward an improvement in public attitudes between 2013/2014 and 2019 in Czechia. While it demonstrates that people exposed to either a nation-wide anti-stigma campaign or to mental health care reform have better attitudes than their nonexposed counterparts, further research is needed to strengthen causal inference in respect to both. Ongoing effort is needed to improve population desire for future contact and address attitudes that have not improved yet.

## Data Availability

Data are available upon a reasonable request, which should be addressed to the corresponding author.
